# Sensitivity of the Kβ″ X-ray Emission Line to Coordination Changes in GeO_2_ and TiO_2_

**DOI:** 10.1021/acs.jpclett.3c00017

**Published:** 2023-02-13

**Authors:** G. Spiekermann, Ch. J. Sahle, J. Niskanen, K. Gilmore, S. Petitgirard, C. Sternemann, J. S. Tse, M. Murakami

**Affiliations:** † ETH Zürich, Rämistrasse 101, 8092 Zürich, Switzerland; ‡ European Synchrotron Radiation Facility (ESRF), 71 Avenue des Martyrs, 38000 Grenoble, France; § Department of Physics and Astronomy, University of Turku, 20014 Turun yliopisto, Finland; ∥ Physics Department and IRIS Adlershof, Humboldt Universität zu Berlin, Zum grossen Windkanal 2, 12489 Berlin, Germany; ⊥ Technische Universität Dortmund, Fakultät Physik/DELTA, Maria-Goeppert-Mayer-Strasse 2, 44227 Dortmund, Germany; # Department of Physics and Engineering Physics, University of Saskatchewan, Saskatoon S7N 5E2, Canada

## Abstract

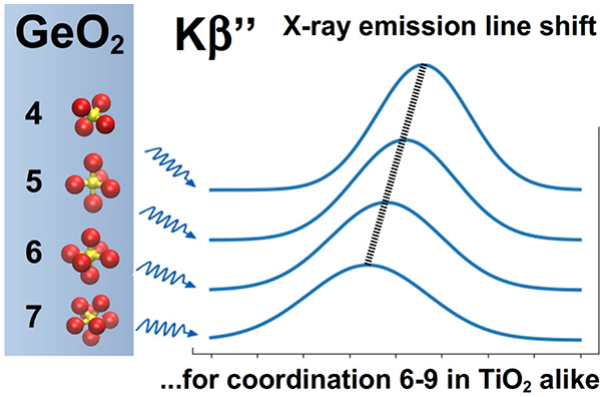

The hard X-ray Kβ″ emission line shows sensitivity with respect to a wide range of cation–ligand coordination, which we investigate in the cases of GeO_2_ and TiO_2_ on the basis of ab initio spectral calculations on amorphous and crystalline structures. In compressed amorphous GeO_2_, the sampling of a large number of instantaneous coordination polyhedra from an ab initio molecular dynamics trajectory reveals that the functional relation between the Kβ″ shift and coordination is close to linear between 4-fold and 7-fold coordination. A similar sensitivity of the Kβ″ emission line exists in the coordination range between six and nine of crystalline high-pressure TiO_2_ polymorphs. Our results demonstrate the potential of the Kβ″ emission line in research on the structure of amorphous oxide material.

Coordination changes are of interest in high-pressure research, where the average cation coordination in various amorphous compounds like GeO_2_, TiO_2_, and SiO_2_ at extreme pressures has received considerable attention in the past years but is challenging to access experimentally.^1−6^ In these systems, the cations undergo an increase of average coordination number (CN) with pressure, from four to six or above in the case of germanium and silicon and from six to nine or above in the case of titanium oxide.^1−3,6,7^ However, there are contradicting results between different studies concerning the onset of average coordination above six in the case of silicon oxide and its analogue germanium oxide.^2−5^ This situation calls for validation of novel approaches, for which we study the potential of the Kβ″ X-ray emission line as a probe. The cation Kβ″ X-ray emission line is one of the weakest features in valence-to-core X-ray emission spectroscopy (vtc XES) of chemical systems with cation–ligand (or metal–ligand) bonds.^8^ It reflects the valence orbital hybridization with the 2s orbitals of the coordinating ligands, as evidenced in projected electronic density-of-states (pDOS) calculations.^5,9−11^ The Kβ″ line is also termed “crossover” transition and is absent in any vtc XES spectrum of a cation in its elemental form. It is known to allow for insight into several aspects of the first ligand coordination shell around the probed atom, such as chemical sensitivity, cation–ligand bond length, and—the target of this study—CN.^8,9,11−25^

The *chemical* sensitivity of the Kβ″ line with respect to the type of ligand results from differences in binding energy of the *n*s electrons of the ligands, e.g., about 5 eV between nitrogen and oxygen, and has been demonstrated in a variety of studies on e.g. Ti, V, Mn, Fe, Cr, Cu, and Nb.^8,9,11−25^ The sensitivity of the Kβ″ line with respect to cation–ligand *bond length* has been demonstrated in e.g. Mn, V, Ti, and Ge systems^5,12−14,23^ and results from the variable extent of overlap between the ligands’ *n*s orbitals with the valence orbitals of the cation: When the bond length shortens, the overlap increases, leading to a higher “crossover” transition probability and therefore to a more intense Kβ″ line.^8^ Its sensitivity with respect to *coordination* has been discussed for a narrow range of coordination states for nickel, iron, and copper,^11,26,27^ but investigations of a wider coordination range are scarce.^5^

In this work we validate Kβ″ spectroscopy as a probe for an extended range of coordination states: First, we quantitatively assess the functional relation between the Kβ″ line shift and coordination in the case of compressed amorphous GeO_2_. Second, we qualitatively investigate the applicability of the Kβ″ line shift to different crystalline high-pressure polymorphs of TiO_2_. Our methods are the ab initio computation of vtc XES spectra of cations in known coordination states using the OCEAN code^28,29^ and their evaluation in terms of the Kβ″ line shift.

GeO_2_ is well-suited to quantitatively investigate the Kβ″ emission line shift over a wide range of coordination, for two reasons: First, the Kβ vtc XES spectra of germanium oxide show a free-standing Kβ″ line, well separated from the neighboring Kβ_5_ and Kβ_2_ emission lines. Second, gradually compressed amorphous GeO_2_ covers coordination states between 4-fold at ambient pressure and 6-fold at about 20 GPa. Furthermore, the Kβ_5_ line serves as inherent calibration reference because its emission energy is not affected by any structural change: The filled and contracted semicore 3d orbitals in germanium oxide follow the trend of the germanium 1s energy level so closely that the resulting emission energy remains constant within experimental uncertainty (see experimental data in Figure 1a, taken from ref (5)). Thus, if considered relative to the energy of the Kβ_5_ line, the Kβ″ line shift can be evaluated from spectra without the need of a precise XES energy calibration.^5^

**Figure 1:**
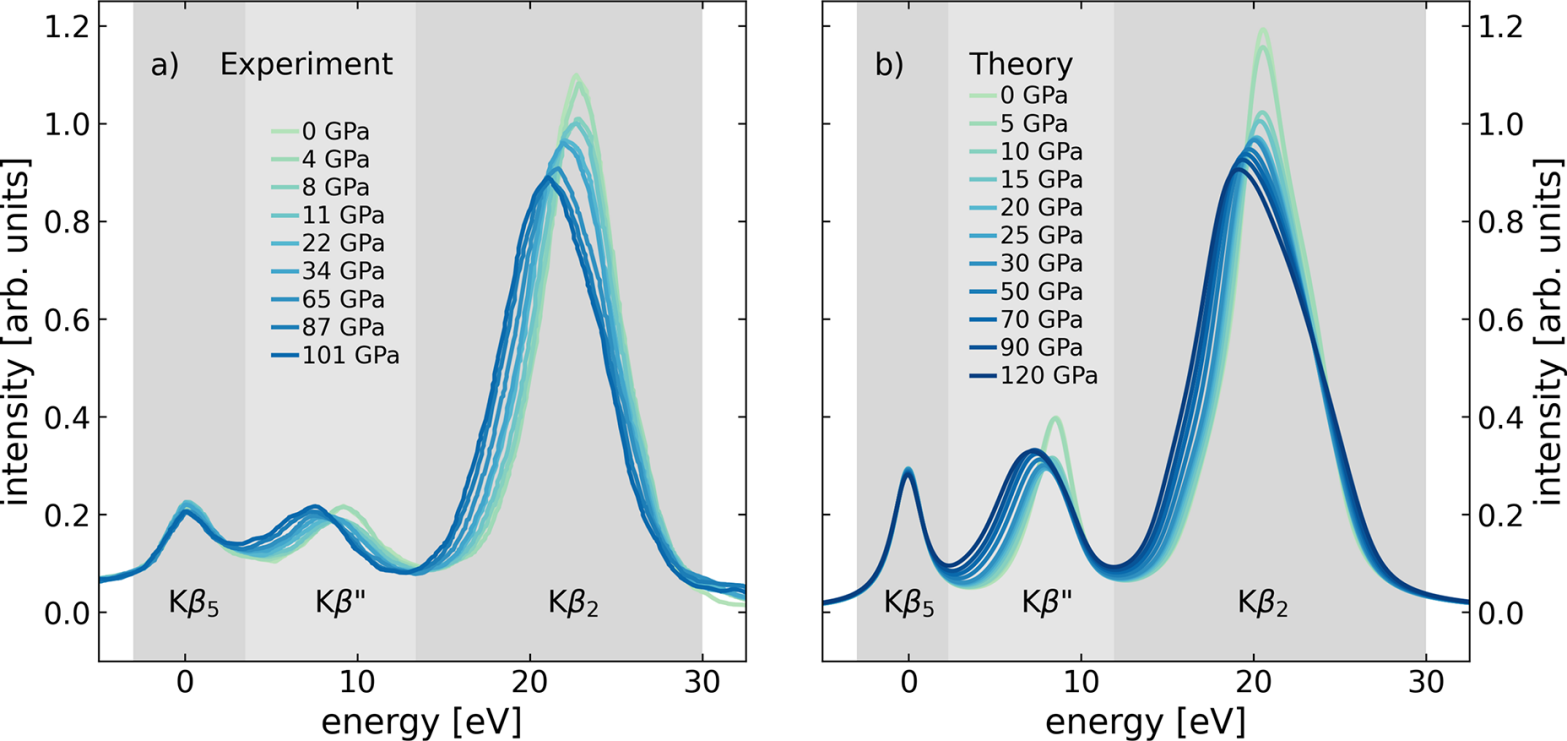
(a) Experimental Kβ valence-to-core X-ray emission spectra of compressed amorphous GeO_2_ from inside a diamond anvil cell at pressures between ambient and 101 GPa.^5^ (b) Calculated spectra of compressed amorphous GeO_2_. At each pressure step of the ab initio molecular dynamics simulation, averaged spectra over all individual Ge sites are shown, aligned to a common position of the Kβ_5_ emission line.

We performed spectral calculations from a large number of configurations of a previously reported *ab initio* molecular dynamics (AIMD) simulation of amorphous GeO_2_ up to 120 GPa.^1,30^ In this way, the ensemble of constant composition allows to sample a large number and variety of independent coordination polyhedra over a wide range of pressures, covering an extended structural parameter space, e.g., with virtually continuously varying degrees of distortion.^31^ This broad sampling of the phase space of the coordination shell in the case of amorphous GeO_2_ allows for a quantitative evaluation, establishing a functional dependence between Kβ″ line shift and the cation CN.

Such extended sampling of the coordination shell parameters in amorphous GeO_2_ leads to high agreement between computed and experimental spectra: We summed at each pressure between 0 and 120 GPa 360 individual vtc XES spectra (72 Ge atoms in five uncorrelated AIMD configurations) and compare the sum (Figure 1b) to experimental spectra (Figure 1a). Experimental spectra were taken from ref (5). In addition, we also show the measured Kβ_2_ line to better illustrate the quality of the agreement between theory and experiment. The overall line shape and the pressure-induced relative changes of the entire experimental spectrum are well reproduced by the simulated spectra, which lends credibility to the spectral calculations.

Our focus on coordination number, rather than on mean cation–ligand bond length or mean polyhedral bond angle, deserves a brief explanation. In fact, each spectrum may hold information about a variety of parameters of the first coordination shell.^31^ We evaluated all individual Ge spectra, irrespective of pressure, in terms of the Kβ″ line shift (here and in the following expressed as Δ*E*, i.e., the difference in emission energy of the Kβ″ line relative to the constant Kβ_5_ line) and plot Δ*E* against CN, mean cation–ligand bond length, and mean polyhedral bond angle in Figure 2a–c. All three parameters are computed for the oxygen atoms found within the first coordination shells, defined by a sphere of 2.3 Å radius centered around the active Ge site.^1^ The correlation between the Kβ″ emission line shift and CN is evident and on average even close to linear (Figure 2a). From the trends as a function of bond length and angle, it is visible that there are clear correlations when the coordination is taken into account (see black symbols for coordination-specific averages in Figure 2b). However, these coordination-specific trends can be applied to experimental results only after coordination is known. For this reason, we focus here on the dependency of the Kβ″ line shift on coordination. Beyond the scope of this work is the correlation of the Kβ″ emission line *intensity* with bond distance, established in a number of works.^5,12−14,23^

**Figure 2:**
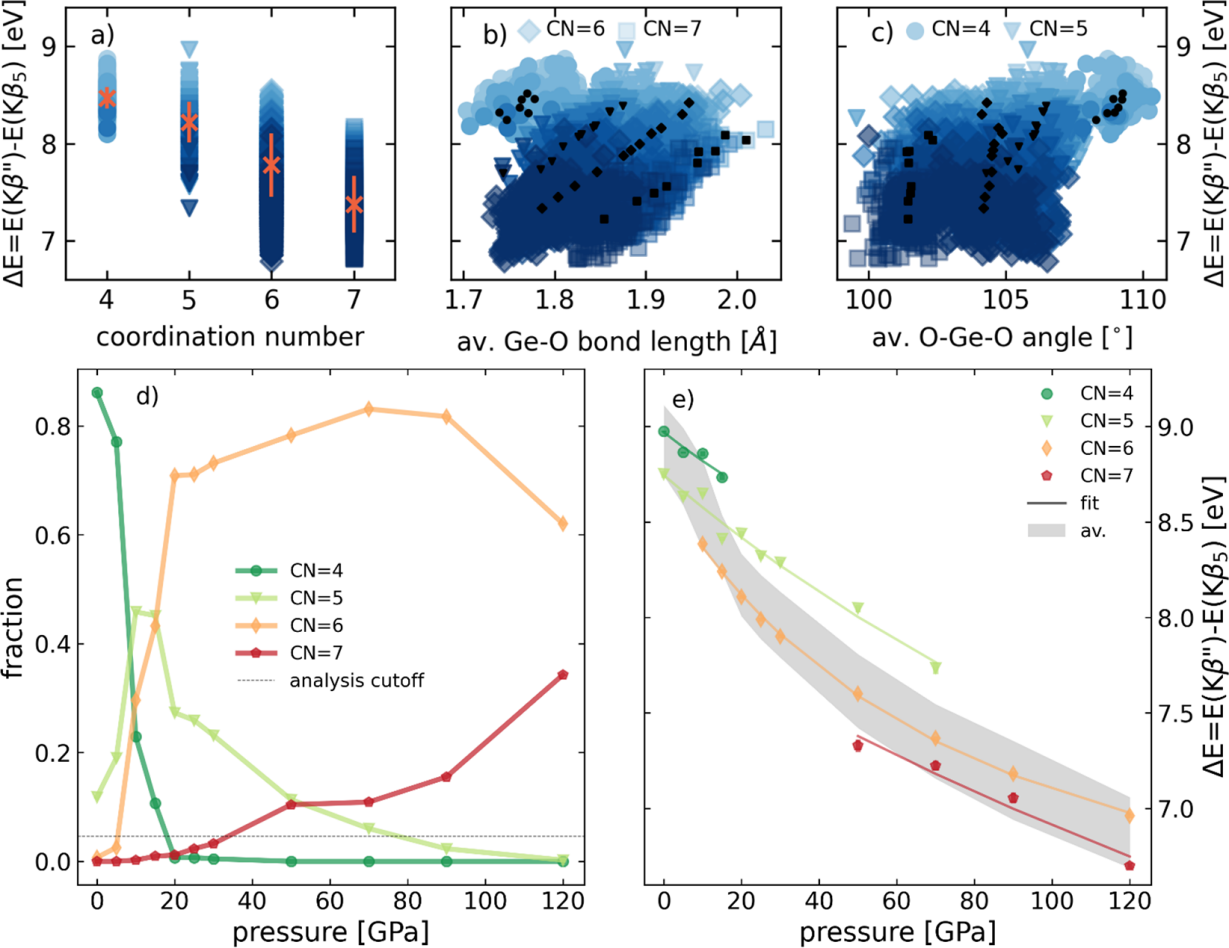
(a–c) Energy difference Δ*E* of the Ge Kβ″ emission line relative to the constant Kβ_5_ line as a function of coordination (a), the average Ge–O bond length (b), and the mean O–Ge–O angle (c). Crosses in (a) denote the average values, and black symbols in (b) and (c) denote coordination-specific averages. (d) Ge–O coordination number analysis of the MD simulation reported by Du et al.^1^ with a radial cutoff of 2.3 Å. (e) Position of the coordination-specific Kβ″ emission line shifting with pressure, expressed as Δ*E* relative to the constant position of the Kβ_5_ line, each trend with an exponential fit (solid lines).

The distribution of coordination states in the simulation is shown in Figure 2d as a function of pressure: 4-fold Ge–O coordination is dominant at pressures up to 20 GPa and 6-fold is dominant at higher pressure, with a fraction of 5-fold present throughout and 7-fold appearing at 50 GPa.^1^ For the correlation between the Kβ″ line shift and CN, at each pressure, we consider only those coordination states that have a relative abundance of at least 5% to ensure a meaningful sampling (dashed horizontal line in Figure 2d), leading to four different coordination states between four and seven.^1^

Next, we investigated the relation between the Kβ″ emission line shift and CN over the entire pressure range. For this, we first averaged a large number of individual spectra of identical CN at each pressure step of the simulation. The resulting coordination-specific dependency of Δ*E* on pressure reveals four trends of similar slope, offset from each other by a few 100 meV (Figure 2e, exponential decay fits indicated by lines). These offsets between the coordination-specific trends of Δ*E* are evidence for the sensitivity of the Kβ″ line with respect to coordination. With information about the pressure-dependent magnitude of these offsets between each coordination-specific pressure trend, the average coordination number in amorphous GeO_2_ can be determined from two parameters: the pressure of the system and the position of the Kβ″ line, which can be determined in experimental spectra with an uncertainty of <100 meV. For illustration, the computed total trend with pressure of Δ*E* of all germanium atoms (shown as a gray trend in Figure 2e, its width of about 0.4 eV representing the scatter of all computed spectra at a given pressure) is the experimentally accessible quantity, from which the average coordination can be inferred, when the coordination-specific trends are known.^5^

As the coordination-specific trends in the case of amorphous GeO_2_ have slightly different slopes (Figure 2e), the offsets between the coordination-specific trends of Δ*E* vary with pressure. We elucidate this pressure dependence by calculating Δ*E* as a function of coordination for ten pressure points between 0 and 100 GPa separately (Figure 3a), yielding curves equivalent to vertical cuts through the fits shown in Figure 2e and their extrapolation. Normalized to the mean Kβ″ shift and averaged over all pressures, the data show a close-to-linear shift of the Kβ″ line with coordination (dashed line in Figure 3a).

**Figure 3:**
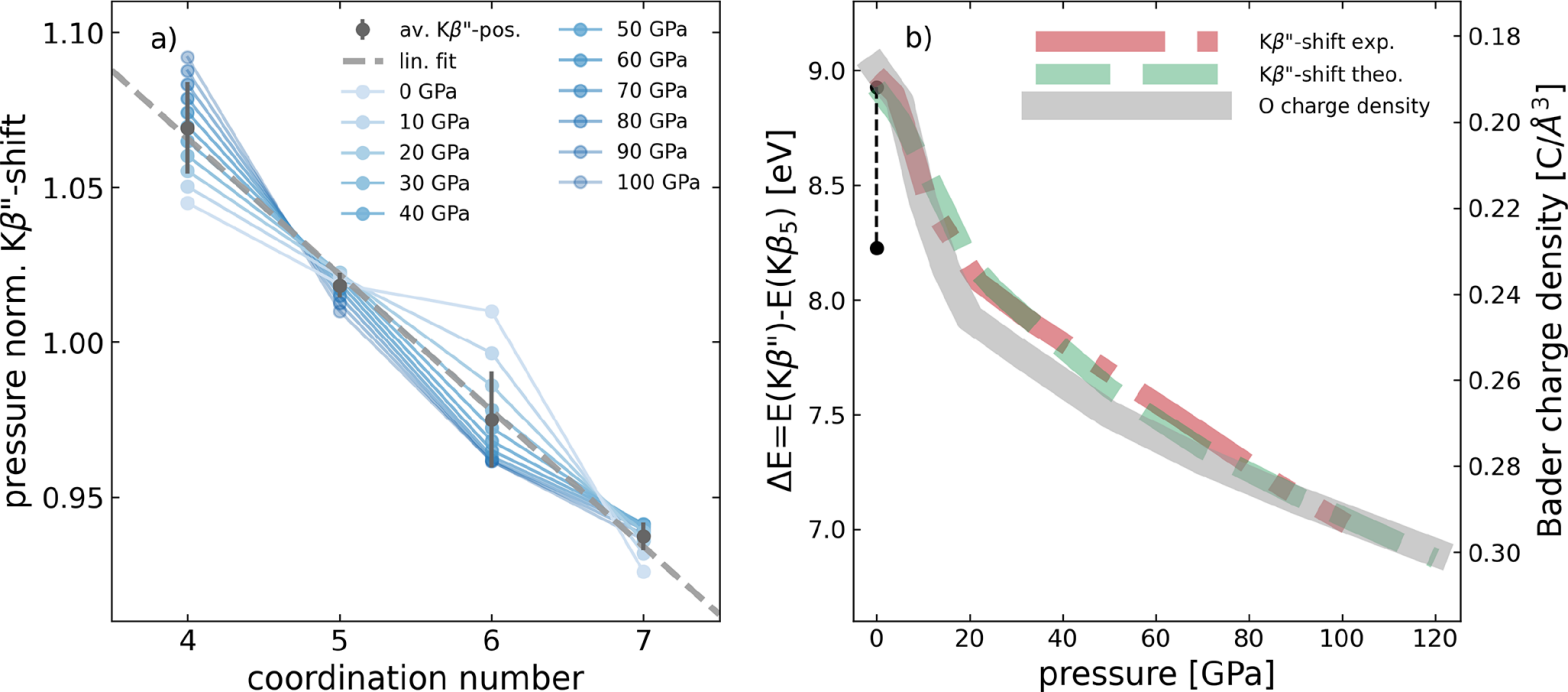
(a) Normalized shift of the Kβ″ emission line as a function of coordination, at each pressure step separately (see text for details). Data points are taken from the fits shown in Figure 2e, partially extrapolated to higher or lower pressure. On average, the correlation follows a linear trend (dashed line). (b) Comparison between the shift of the Kβ″ line and the Bader charge density at the oxygen sites (left and right *y*-axis, respectively) as a function of pressure. The black dumbbell marks the measured difference of 0.7 eV of the Kβ″ emission line position between GeO_2_ with 4- and 6-fold coordination at ambient pressure.^5^

The origin of the Kβ″ line shift, although not the main focus of this study, deserves some attention. The previously computationally observed increased oxygen charge density in compressed amorphous GeO_2_ and SiO_2_ has been interpreted as increasing ionic character of these compounds.^1^ Also on the basis of charge density, an increase in ionicity had been stated before for the transition from 4-fold to 6-fold coordination in crystalline GeO_2_ and SiO_2_ compounds of quartz- and rutile-like structure at ambient pressure.^32,33^

To align with these previous observations, we computed the oxygen Bader charge density and show its average at each pressure as gray curve in Figure 3b (right *y*-axis). It is found to increase monotonically with pressure, with a strong increase of charge density at the oxygen site by about 30%  in the pressure range from 0 to about 20 GPa, in which the coordination increases from 4-fold to predominantly 6-fold. Because of the general volume compression, also the charge density at the Ge sites increases (not shown), but considerably less in both absolute and relative values. Interestingly, the evolution with pressure of the shift of the Kβ″ line to lower emission energy correlates with the increase of charge density at the oxygen site over the entire pressure range (Figure 3, left *y*-axis). Furthermore, the measured Kβ″ line shift at ambient pressure of 0.7 eV between the quartz-like structure and the rutile-like structure (shown as black dumbbell mark in Figure 3b) anticipates the coordination-change induced high-pressure trend up to about 20 GPa.

X-ray emission spectroscopy, rather than recording absolute shifts, records the difference between the 1s core level and valence levels. When both levels shift the same amount of energy, e.g., due to change in Madelung potential, there is no observable shift. This is the case for the contracted Ge 3d states, whose transition line Kβ_5_ remains constant in emission energy across all recorded spectra. The Kβ″ shift of 0.7 eV at ambient pressure, due to increased coordination at the simultaneous 8% increase of bond length, shows that coordination is the strongest single factor influencing the Kβ″ shift. A difference in relative population of germanium 4s and 4p orbitals, both hybridized with oxygen 2s, is thus probably the strongest factor causing the observed Kβ″ shift, followed by bond shortening. The accompanying contraction of the volume leads to the correlation of the oxygen charge density and the Kβ″ line shift as shown in Figure 3b.

The second aim of our study is to demonstrate qualitatively the potential of our approach to study other cations and higher CN range. This is illustrated on TiO_2_. TiO_2_ is a good choice for two reasons: First, the Kβ″ emission line is free-standing in the vtc XES spectrum of titanium compounds, in calculation and experiment separated from the neighboring Kβ_1,3_ and Kβ_2,5_ lines (Figure 4a).^13,14,17,20−23,25^ Second, the crystalline high-pressure polymorphs of TiO_2_ cover an unusual and wide range of coordination states: The crystalline high-pressure TiO_2_ polymorphs are rutile-type, ZrO_2_-type (baddeleyite), and PbCl_2_-type (cotunnite) structures, with 6-, 7-, and 9-fold coordination of titanium, respectively.^34,35^ A crystalline structure of titanium in 8-fold coordination has not been found experimentally, to the best of our knowledge.

**Figure 4:**
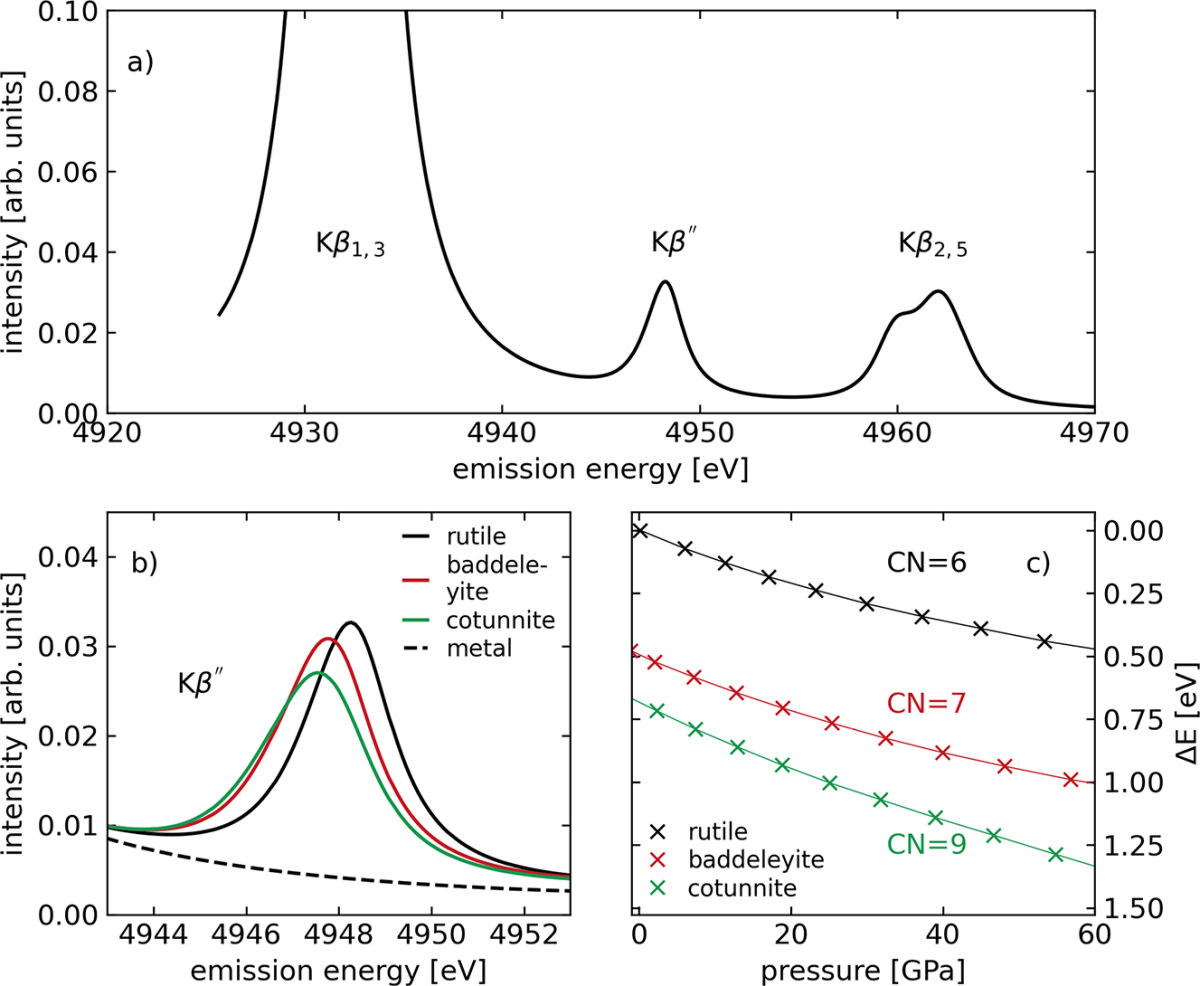
(a) Computed vtc XES spectrum of rutile-TiO_2_. (b) Zoom into the Ti Kβ″ emission line of calculated vtc XES spectra of TiO_2_ polymorphs: rutile, baddeleyite, and cotunnite structure of 6-, 7-, and 9-fold coordination, respectively, all near ambient pressure. The spectrum of metallic titanium is shown for reference (dashed line). (c) shift of the titanium Kβ″ emission line of these polymorphs as a function of pressure, as obtained in the computed spectra, expressed as Δ*E* relative to the Kβ″ line position of rutile-structured TiO_2_ at ambient pressure.

Our calculated vtc XES spectrum of rutile-TiO_2_ is in reasonable agreement with experimental data (see, e.g., refs (23 and 25)) and shows the Kβ_1,3_, Kβ″, and Kβ_2,5_ lines separated from each other (Figure 4a). The simulated spectra of the three TiO_2_ polymorphs of the rutile-, ZrO_2_-, and PbCl_2_-type with 6-, 7-, and 9-fold coordination (and of metallic titanium as reference) are shown in Figure 4b, normalized to the area of the Kβ_1,3_ line. The Kβ″ line shifts from one polymorph to the other by a few 100 meV at ambient pressure, while being absent in the case of metallic Ti, as expected.

Next, we compressed the unit cells of the three polymorphs in steps and computed the vtc XES spectrum and the pressure at each pressure. This allowed us to assess the coordination-specific Kβ″ line shift as a function of pressure (Figure 4c). Here we consider its shift as relative to the position in rutile-TiO_2_ at ambient pressure, in lack of an inherent calibrating emission line as the Kβ_5_ line in the case of GeO_2_.^36^

The Kβ″ line shift between the different TiO_2_ polymorphs at ambient pressure is persistent at high pressure and amounts to about 0.5 eV between 6- and 7-fold coordinated titanium and to 0.3 eV between 7- and 9-fold coordinated titanium. The functional relation between coordination and the Kβ″ line shift thus is not linear in this case of strictly crystalline structures of TiO_2_. The spectra of crystalline structures lack a sampling of the parameter space of the first coordination shell and may therefore be biased.^37^ However, the trends in Figure 4 are encouraging to further investigate this dependency in the case of amorphous titanium compounds.^6^

There is a number of elements to which the Kβ″ emission line evaluation can be applied as done here. We hypothesize that the approach is not limited to the oxides of a single cation: With appropriate reference samples and computational support, oxides with several cations could be investigated, allowing for special insight, as diffraction-based techniques can reach their limit when investigating coordination in multicomponent oxides. This approach can provide a unique means of structural investigation of chemically complex, amorphous material at high pressure or in other confined sample environments.

In summary, we have elucidated quantitatively the sensitivity of the Kβ″ emission line with respect to an extended range of coordination states in the case of compressed amorphous GeO_2_. Pressure as a canonical variable and the extensive sampling of the structural parameters in the AIMD simulation of amorphous GeO_2_ allowed us to quantify the shift observed in the Kβ″ line of Ge as a function of coordination state beyond structural bias. The functional relation between the position of the Kβ″ line and the cation–ligand coordination between four and seven was found to be nearly linear. The origin of the shift lies primarily in a relative change in the hybridization between oxygen 2s and metal cation valence orbitals, and to a lesser extent in the amount of overlap, increased by shortening of bonds. Computed spectra of high-pressure TiO_2_ polymorphs reveal a consistent sensitivity with respect to even higher coordination in the range between six and nine. This approach can provide new insight into the debated coordination evolution in compressed amorphous oxides. The application to chemically more complex oxides seems possible but needs additional investigation.
